# Underlying causes for prevalent false positives and false negatives in STARR-seq data

**DOI:** 10.1093/nargab/lqad085

**Published:** 2023-09-22

**Authors:** Pengyu Ni, Siwen Wu, Zhengchang Su

**Affiliations:** Department of Bioinformatics and Genomics, The University of North Carolina at Charlotte, Charlotte, NC 28223, USA; Department of Bioinformatics and Genomics, The University of North Carolina at Charlotte, Charlotte, NC 28223, USA; Department of Bioinformatics and Genomics, The University of North Carolina at Charlotte, Charlotte, NC 28223, USA

## Abstract

Self-transcribing active regulatory region sequencing (STARR-seq) and its variants have been widely used to characterize enhancers. However, it has been reported that up to 87% of STARR-seq peaks are located in repressive chromatin and are not functional in the tested cells. While some of the STARR-seq peaks in repressive chromatin might be active in other cell/tissue types, some others might be false positives. Meanwhile, many active enhancers may not be identified by the current STARR-seq methods. Although methods have been proposed to mitigate systematic errors caused by the use of plasmid vectors, the artifacts due to the intrinsic limitations of current STARR-seq methods are still prevalent and the underlying causes are not fully understood. Based on predicted *cis*-regulatory modules (CRMs) and non-CRMs in the human genome as well as predicted active CRMs and non-active CRMs in a few human cell lines/tissues with STARR-seq data available, we reveal prevalent false positives and false negatives in STARR-seq peaks generated by major variants of STARR-seq methods and possible underlying causes. Our results will help design strategies to improve STARR-seq methods and interpret the results.

## INTRODUCTION

*cis*-Regulatory modules (CRMs) formed by closely located clusters of transcription factor (TF) binding sites (TFBSs) are as important as coding DNA sequences in specifying complex traits of animals (1,2). It has been shown that most (95.4%) of genetic variants associated with human complex traits and diseases are located in non-coding sequences (3–5), and mostly likely in CRMs (6–13). Non-coding variants in a CRM can alter the TF binding affinity, leading to epigenomic changes in relevant cells (14,15), and eventually affecting organismal phenotypes (16). However, linking non-coding variants to complex phenotypes is challenging, due to our limited knowledge of all CRMs and their constituent TFBSs (17). Thus, it is essential to categorize all CRMs and constituent TFBSs in the genomes and determine their functional states and target genes in various cell types in humans and other organisms (17). A CRM often functions independently of its location and orientation relative to the target genes (18). A CRM regulates the transcription of a target gene through interactions between the TFBSs in the CRM and cognate TFs in certain cell types, where the former are accessible and the latter are available to bind (18,19). Enhancers that increase the expression of target genes are arguably the most important and best-studied type of CRMs. Although more than a million CRMs have been predicted in the human genome, only a few thousand of them have been experimentally validated as enhancers using reporter assays in transgene animal models (20).

More recently, various massively parallel reporter assays (MPRAs) have been developed to characterize and validate enhancers (21). In particular, one type of MPRAs, self-transcribing active regulatory region sequencing (STARR-seq), originally developed for small genomes, clones randomly sheared DNA segments between a minimal promoter-driven green fluorescence protein open reading frame and a downstream polyA sequence (22). If a sequence is an active enhancer, this results in the transcription of the enhancer sequence, allowing us to assess enhancer activities of potentially all DNA segments in the genome. STARR-seq and its variants such as WHG-STARR-seq (23), ATAC-STARR-seq (24), ChIP-STARR-seq (25) and CapSTARR-seq (26) have been applied to mammalian cells, and a lot of insights into enhancers have been gained from these studies (25,27–30). However, two intrinsic limitations of episomal expression vectors used in these methods may lead to artifacts (22–25,31–34). First, since candidate sequences in expression vectors lack the native chromosomal context, a large portion of identified STARR-seq peaks (STARR peaks) are silenced in tested cells. For example, it has been shown that 31%, 52% and 87% of STARR peaks identified in fly S2 cells (22), human GM12878 cells (24) and LNCaP cells (23), respectively, are in native repressive chromatin. Although the STARR peaks identified in repressive euchromatin of tested cells might be *bona fide* enhancers that could be active in other cell/tissue types, those found in heterochromatin might be false positives. Second, usually only short (<500 bp) candidate sequences can be efficiently inserted in the plasmids in most STARR-seq methods; since most known enhancers are longer than 500 bp (20), the vast majority of active enhancers can be missed by these STARR-seq methods (31,34), resulting in high false negative rates. Recently, it was also reported that the pGL3/4 plasmid that was widely used in the STARR-seq protocols had two systematic errors (31). First, the origin of replication site (ORI) of the plasmid can function as a strong core promoter to initiate transcription, thereby interfering with the transcription from the core promoter of the report gene, resulting in false negative results (31). Second, transfection of the plasmid in certain human cells can induce a strong type I interferon (IFN-I) response and other irrelevant transcriptions, leading to false positive results (31). It was found that using ORI as the core promoter of the reporter gene and, at the same time, repressing the IFN-I response by specific inhibitors could largely reduce these errors (31). Although this strategy has been widely adopted, it does not circumvent the two aforementioned inevitable intrinsic limitations of plasmid expression systems. Thus, the artifacts of the methods remain. Nonetheless, the prevalence of the artifacts is unknown, and the underlying causes are not fully understood. Based on our recently predicted CRMs and non-CRMs with high accuracy in 85% of the human genome (35) as well as our predicted active enhancers and non-active CRMs (35) in multiple human cell lines/tissues to which various STARR-seq methods have been applied, we addressed these important issues.

## MATERIALS AND METHODS

### The datasets

We downloaded from the database of predicted *cis*-regulatory modules (http://cci-bioinfo.uncc.edu) (36) 1 426 947 CRMs and 1 755 876 non-CRMs predicted in 85% of the human genome regions using the dePCRM2 pipeline (35,37). We downloaded the silencer list from SilencerDB (38) and CTCT peaks from Cistrome Data Browser (39). We downloaded from the ENCODE data portal (https://www.encodeproject.org/functional-characterization-experiments/) the WHG-STARR-seq (40) peaks in A549, HCT116, HepG2, K562 and MCF-7 cell lines, and CapSTARR-seq (26) peaks in pancreatic organoid tissues. We downloaded ATAC-STARR-seq peaks in the GM12878 cell line from (24) and the STARR-seq peaks in HeLaS3 with IFN-I response inhibitor treatment (HeLa3-I) and without IFN inhibitor treatment (HeLa3-N) from (31). If there were replicates of data in a cell line, we used the overlapping STARR peaks in the replicates to generate a unique STARR-seq dataset. All these STARR-seq datasets were generated using ORI as the core promoter of the reporter gene, except for those in GM12878 where the SPC1 promoter was used (24). More details about the datasets are summarized in Supplementary Table S1. To exclude possible silencers and insulators from our analysis, we filtered out the CRMs that overlap with the downloaded silencers or CTCF peaks if at least 50% of their respective lengths overlap reciprocally. Moreover, although some promoters can also function as distal enhancers (41), we excluded possible promoters from our analysis by filtering out predicted CRMs that were located within 1000 bp from the nearest transcription start sites (TSSs) of genes. We downloaded from the ENCODE data portal RNA-seq data in A549, HCT116, HepG2, K562, MCF-7, GM12878, HeLaS3-I, HeLaS-N and pancreatic organoid cell lines/tissues. We downloaded from the Cistrome database (39) the epigenetic marks [chromatin accessibility (CA) (assay for transposase-accessible chromatin using sequencing, ATAC-seq), H3K4me1, H3K27ac, H3K4me3, H3K9me3 and H3K27me3] in the cell lines/tissues. For validation, we downloaded the ChromHMM expanded 18-state annotations in seven cell lines/tissues (A549, GM12878, HepG2, K562, HeLaS3-I, HeLaS3-N and pancreatic organoid) from Roadmap Epigenomics Consortium (42) and the transposal elements in the human genome from UCSC Genome Browser (43).

### Prediction of active and non-active CRMs

We used our previously trained logistic regression-based universal functional state predictor (UFSP) to predict the enhancer functional state (active or non-active) of all the CRMs (with potential silencers and insulators excluded) in the cell lines/tissues using signals of four active enhancer epigenetic marks CA (ATAC-seq), H3K4me1, H3K4me3 and H3K27ac, as previously described (35). Briefly, we trained the UFSP on a negative set made up of all the predicted non-CRMs in the human genome and a positive set made by pooling the positive set in each of 67 human cell lines/tissues (35). A positive set in a cell/tissue type consists of the CRMs that overlap at least a binding peak of TF ChIP-seq datasets collected in the cell/tissue types (35). The signals of the four epigenetic marks CA (ATAC-seq), H3K4me1, H3K4me3 and H3K27ac on the sequences were used as the features in UFSP.

### Association of sequence elements to genes

As accurate prediction of target genes to distal CRMs is still a highly challenging task, and the existing methods are not necessarily superior to the simple ‘closest gene assignment’ (44), we assigned the gene closest to a sequence element as the target of the latter.

### Defining genome categories

**Figure 1.**
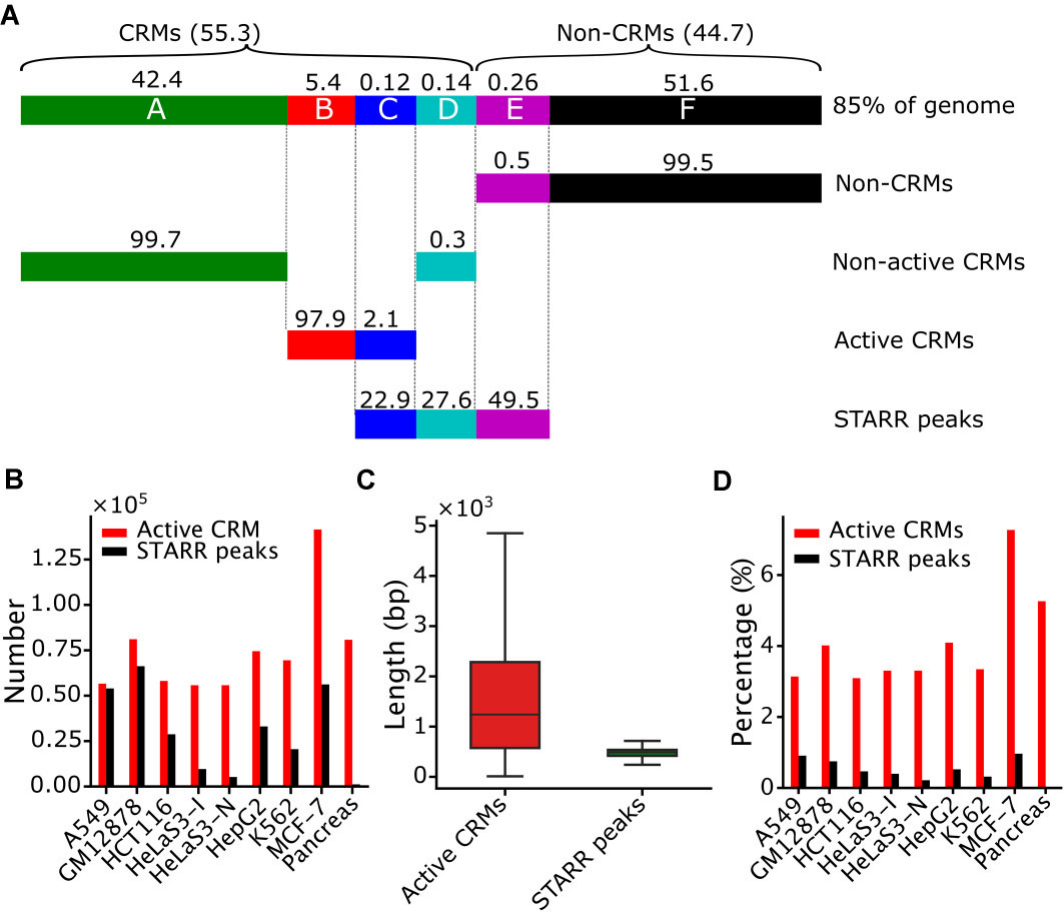
Comparison of our predicted active CRMs and STARR peaks. (**A**) Cartoon showing that positions of the 85% of genome regions in a cell line can be exclusively divided into six categories A–F based on overlaps among predicted active CRMs, predicted non-active CRMs and STARR peaks in a cell line as well as predicted CRMs and non-CRMs in the genome (see the ‘Materials and Methods’ section for the definitions of the categories). The number above a box is the mean percentage of the indicated category in the nine cell lines/tissues. For clarity, the lengths of lines are not proportional to the numbers; for insight into each category in each cell line/tissue, see Supplementary Figure S10. (**B**) Numbers of STARR peaks and predicted active CRMs in each cell line/tissue. (**C**) Distributions of the lengths of STARR peaks and predicted active CRMs in pooled nine cell lines/tissues. (**D**) Percentages of genome positions covered by STARR peaks and predicted active CRMs in each cell line/tissue.

In each cell/tissue, we divided each nucleotide position in 85% of the human genome from which we were able to predict CRMs and non-CRMs into one of six categories A–F, based on whether the position falls in a CRM or a non-CRM, overlaps with a STARR peak or not in the cell/tissue, and falls in an active CRM or a non-active CRM in the cell/tissue type (Figure 1A). Specifically, category A consists of nucleotide positions of non-active CRMs that do not overlap the STARR peaks, category B consists of positions of active CRMs that do not overlap the STARR peaks, category C consists of positions of active CRMs that overlap the STARR peaks, category D consists of positions of non-active CRMs that overlap the STARR peaks, category E consists of positions of non-CRMs that overlap the STARR peaks and category F consists of positions of non-CRMs that do not overlap the STARR peaks (Figure 1A). Moreover, we define that a STARR peak overlaps with a CRM if they overlap at least 50% of their respective lengths reciprocally (37).

### CRM/STARR peak structure complexity

To measure the structural complexity of a CRM or a STARR peak, we mapped our previously predicted TFBSs (37) into the CRM or the STARR peak and calculated the average degree of the TFBSs’ corresponding unique motifs in our previously constructed motif interaction network (37). We then defined the complexity of a CRM or a STARR peak as follows:


(1) \begin{eqnarray*}{\rm complexity} = \log ({N_{{\rm TFBS}}} \times D + 1),\end{eqnarray*}

where ${N_{{\rm TFBS}}}$ is the number of TFBSs per 100 bp in the CRM or STARR peak and *D* is the average degree of the TFBSs’ corresponding unique motifs in the interaction network.

### Enrichment analysis of ChromHMM states and transposable elements

We randomly selected DNA sequences from the whole genome with the matched number and length distribution of the CRMs/STARR peaks in categories A–E. We defined enrichment as follows:


(2) \begin{eqnarray*}{\rm fold}\,{\rm change} = ({{N - M}})/M.\end{eqnarray*}

For ChromHMM state (43) enrichment, *N* is the total length (bp) of CRMs/STARR peaks covered by a state and *N* is the total length of randomly selected sequences covered by the state. For TE enrichment, *N* is the number of TEs in the CRMs/STARR peaks and *M* is the number of TEs in randomly selected sequences.

### Construction of heatmaps of various chromatin signals

To make the heatmap and density plots, we used all CRMs in category C and all STARR peaks in categories D and E. We randomly sampled 10 000 CRMs in category A and B regions. Moreover, we sampled the same number of non-CRMs in category F as the STARR peaks in category E. We extracted a 6 kb segment of genome DNA centered on the middle of each sequence, and for each 100 bp sliding window, we calculated the mean of fold enrichment of each chromatin signal (normalized by library size) to input signal (normalized by library size) using EnrichedHeatmap (w0 mode) (45).

### Statistical tests

A two-tailed Mann–Whitney *U* test was used to compare the gene expression levels, GC content, CRM/STARR peak lengths and CRM complexity. The Kolmogorov–Smirnov (K-S) test was used to compare the distributions of data (density graphs).

## RESULTS

Based on combinatory patterns of TFBSs found in 11 348 TF ChIP-seq datasets for 1360 TFs in 722 cell/tissue types, whose 1000 bp binding peaks cover 85% of the human genome, we predicted a DNA segment in the genome regions to be either a CRM or a non-CRM, thereby partitioning the genome regions into two exclusive sets: 1.43M CRMs and 1.76M non-CRMs (35,37) (Figure 1A). After filtering possible silencers, insulators and promoters (see the ‘Materials and Methods’ section), we ended up with a total of 1 078 604 CRMs as enhancers. We predicted each of these remaining CRMs to be either active or non-active in a cell line based on their CA and three active histone marks (H3K4me1, H3K4me3 and H3K27ac) (35), thereby partitioning the CRMs into two exclusive sets in the cell/tissue type, i.e. active CRMs and non-active CRMs (Figure 1A).

### Overlaps between STARR peaks and predicted CRMs and non-CRMs

We predicted roughly the same number of active CRMs as STARR peaks in two cell lines (A549 and GM12878), 68 times more active CRMs than the CapSTARR peaks in the pancreatic organoid and 2–10 times as many active CRMs as the STARR peaks in the remaining six cell lines (Figure 1B). With an average length of 1581 bp, our predicted active CRMs were much longer than the STARR peaks with an average median length of 512 bp in pooled nine cell lines/tissues (Figure 1C and Supplementary Figure S1). This might be due to the size selection step of DNA fragments in constructing expression vector libraries in STARR-seq protocols to cope with the limitation of insertion sizes in the vectors (34,46). Notably, STARR peaks in HeLaS3-I and HeLaS3-N cells are highly uniform, and both with an average length of 1281 bp; they are at least twice as long as those (∼500 bp) in other cell lines/tissues (Supplementary Figure S1) due to a longer insertion size used in library preparation (31). Moreover, our predicted active CRMs in each cell line/tissue cover a much larger portion of the genome (4.1% on average) than STARR peaks (0.5% on average) (Figure 1D). Notably, twice as many STARR peaks were identified in HeLaS3-I cells as in HeLaS3-N cells (Figure 1B) as previously reported (31), and the STARR peaks in HeLaS3-I cells cover twice as high proportion of the genome as those in HeLaS3-N cells (Figure 1D). Thus, treatment of cells with IFN-I response inhibitors could increase the detection of STARR peaks. On the other hand, the much smaller number of STARR peaks identified in the pancreatic organoid than our predicted active CRMs might be due to the limitation of probes used in the CapSTARR-seq method to capture potential enhancer sequences (26).

To see whether we overpredicted active CRMs or STARR-seq methods underdetermined active CRMs, we analyzed the relation of STARR peaks to our predicted active CRMs, non-active CRMs in a cell line/tissue and the non-CRMs in the 85% of human genome regions. The vast majority (98.8% on average) of nucleotide positions of the STARR peaks in a cell line/tissue fell in the 85% of the genome regions, so we ignored the remaining small portion (1.2% on average) that were located in the 15% of the genome regions where we were not able to predict CRMs and non-CRMs due to the lack of TF binding data in these regions (35–37). Based on overlaps of the four sets of sequences (non-CRMs, non-active CRMs, active CRMs and STARR peaks) in a cell line/tissue, we divide the nucleotide positions of the genome regions in the cell line/tissue into six categories (A–F) (Figure 1A; for details, see the ‘Materials and Methods’ section). We validated active CRMs, non-CRMs, non-active CRMs and STARR peaks in these category regions using expression levels of the genes whose TSSs are closest to these elements on linear chromosomes. If a CRM/STARR peak in a category region is closest to a gene’s TSS on a linear chromosome, we assume that the gene is associated with category.

### Only 22.9% of STARR peak positions overlap active CRM positions, while only 2.1% of active CRM positions overlap STARR peak positions

**Figure 2.**
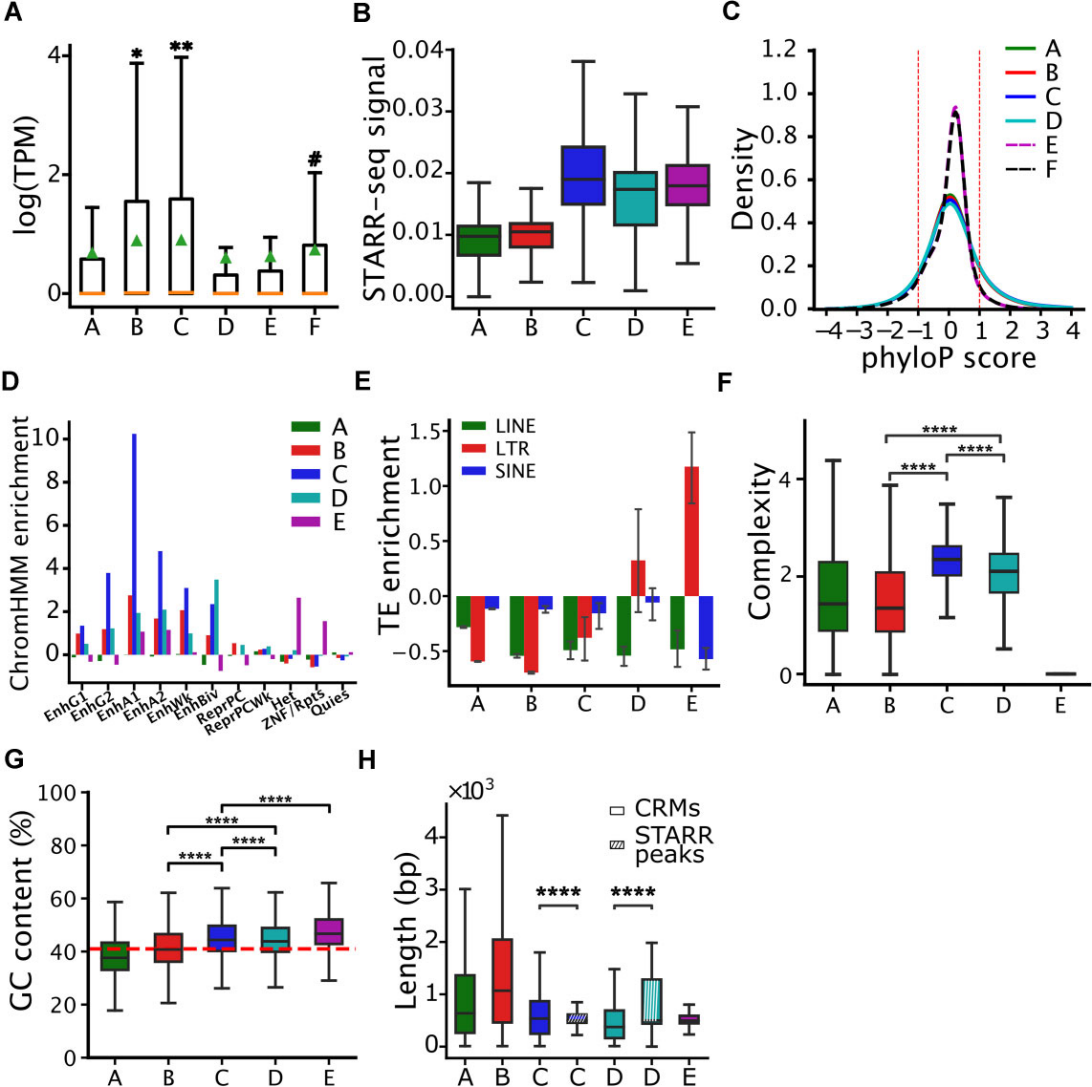
Properties of STARR peaks and predicted CRMs in the six categories. (**A**) Boxplot of expression levels of genes associated with each of the six categories in pooled nine cell lines/tissues. *P< 5.03 × 10^−115^, comparison between genes associated with category B and those associated with categories A, D, E and F. **P< 3.46 × 10^−169^, comparison between genes associated with category C and those associated with the other five categories. ^#^*P*= 2.03 × 10^−27^, comparison between genes associated with category F and those associated with category E. All tests were done using a two-tailed Mann–Whitney *U* test. (**B**) Boxplot of STARR-seq signal strengths in categories A–E in pooled nine cell lines/tissues. (**C**) Distributions of phyloP scores of nucleotide positions of the six categories in pooled nine cell lines/tissues. (**D**) The enrichment of ChromHMM states in categories A–E compared to randomly selected genomic regions. (**E**) The enrichment of transposable elements in categories A–E compared to randomly selected genomic regions. (**F**) Boxplot of CRM complexity of the CRMs/STARR peaks in categories A–E. (**G**) Boxplot GC content of the CRMs/STARR peaks in categories A–E. The dotted horizontal line indicates the mean GC content of 41% in the human genome. (**H**) Boxplot of the lengths of predicted active CRMs and non-active CRMs in categories A–D, and of STARR peaks in categories C–E in pooled nine cell lines/tissues.

**Figure 3.**
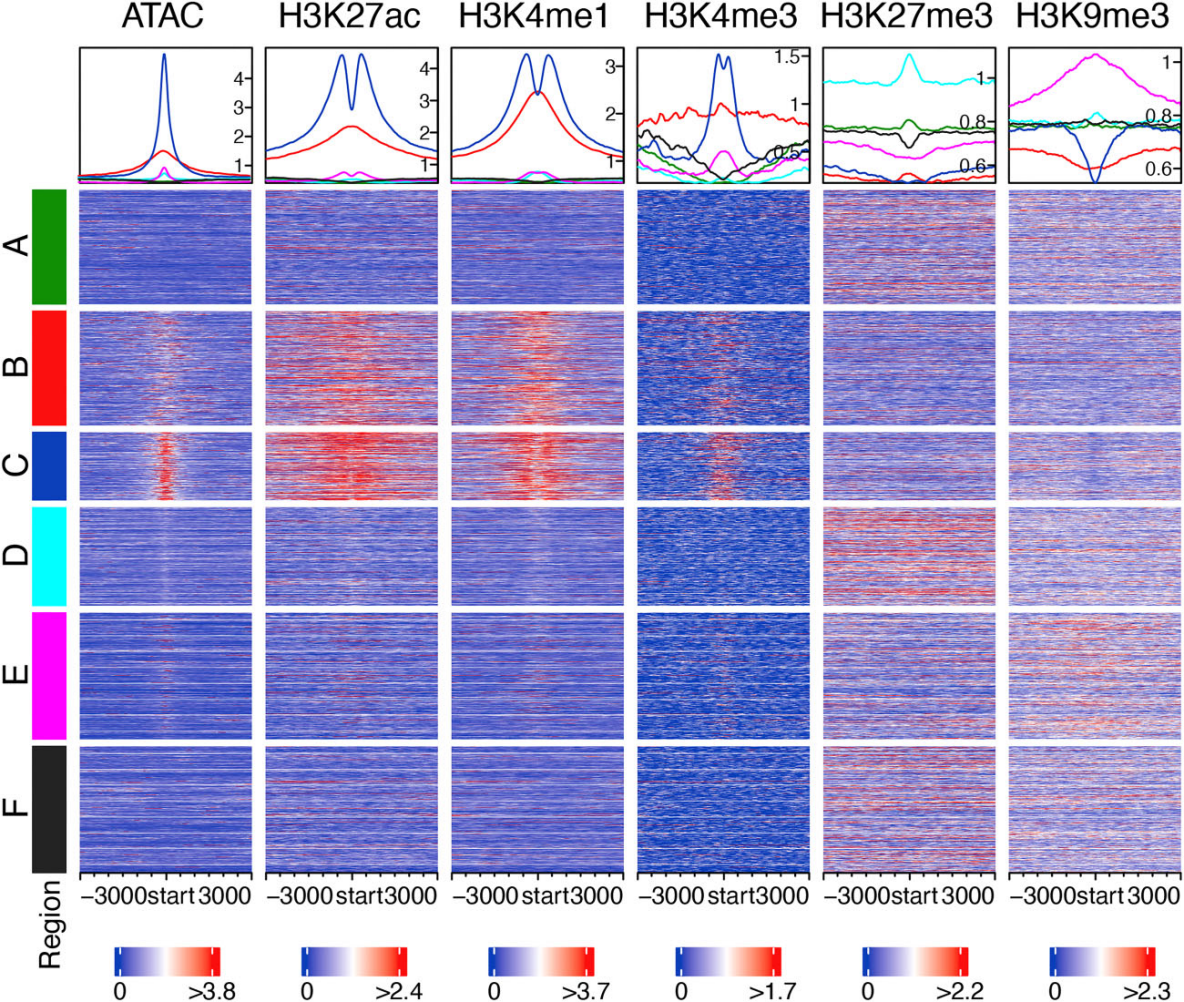
Heatmaps of various chromatin signals in the six categories A–F in the A549 cells. The heatmap shows the mean signal in each window in each sequence and the density plot shows the mean signal of each window position across sampled sequences in the same category (see the ‘Materials and Methods’ section). The color code for category in the density plot above each column is the same as in the heatmaps.

Genes associated with category C (positions of active CRMs that overlap STARR peaks in the cell line) had similarly high gene expression levels to those associated with category B (positions of active CRMs that do not overlap STARR peaks in the cell line/tissue), although they are significantly different (*P*= 2.7 × 10^−5^) in pooled nine cell lines/tissues (Figure 2A) and in seven out of nine cell lines/tissues (Supplementary Figure S2). However, genes associated with category C had significantly higher expression levels (*P*< 3.46 × 10^−169^) than those associated with categories A, D, E and F in pooled nine cell lines/tissues (Figure 2A) and in each of the nine cell lines/tissues (Supplementary Figure S2). Consistently, STARR-seq signals in category C were stronger than those in categories D and E in pooled nine cell lines/tissues (Figure 2B) and in seven of the nine cell lines/tissues (Supplementary Figure S3), indicating that the resulting STARR peaks had higher enhancer activities in STARR-seq assays. These results suggest that the STARR peaks and their overlapping active CRMs in category C were likely to enhance the expression of the closest genes in their native chromatin. As expected, category C is under strong evolutionary constraints (Figure 2C and Supplementary Figure S4), suggesting that the STARR peaks and their overlapping predicted active CRMs are at least parts of true CRMs as we argued earlier (35,37). Consistently, CRMs/STARR peaks in category C were heavily modified by the active chromatin marks of enhancers H3K27ac, H3K4me1 and H3K4me3, and had high CA as measured by ATAC-seq, but were depleted of the repressive enhancer marks H3K9me3 and H3K27me3 (Figure 3 and Supplementary Figure S5). Interestingly, CA signals of the CRMs/STARR peaks in category C are quite narrow, and H3K27ac, H3K4me1 and H3K4me3 signals are relatively depleted in the middle of the CRMs, indicating that TFBSs that are nucleosome-free are concentrated in the middle of the CRMs (Figure 3 and Supplementary Figure S5). Moreover, these active CRMs/STARR peaks are enriched for enhancer states (EnhG1, EnhG2, EnhA1, EnhA2, EnhWk and EnhBiv), but depleted of Het and ZNF/Rpts states in all the seven cell lines/tissues with ChromHMM annotations (Figure 2D and Supplementary Figure S6). They are also depleted of TEs in all the nine cell lines/tissues (Figure 2E and Supplementary Figure S7). They have the highest level of complexity among the five categories A–E analyzed (Figure 2F and Supplementary Figure S8), indicating that they have a higher density of TFBSs whose cognate TFs have more frequent interactions. These CRMs/STARR peaks also have a higher average GC content (45.2%) than the average level (41.0%) of the human genome (Figure 2G and Supplementary Figure S9).

**Figure 4.**
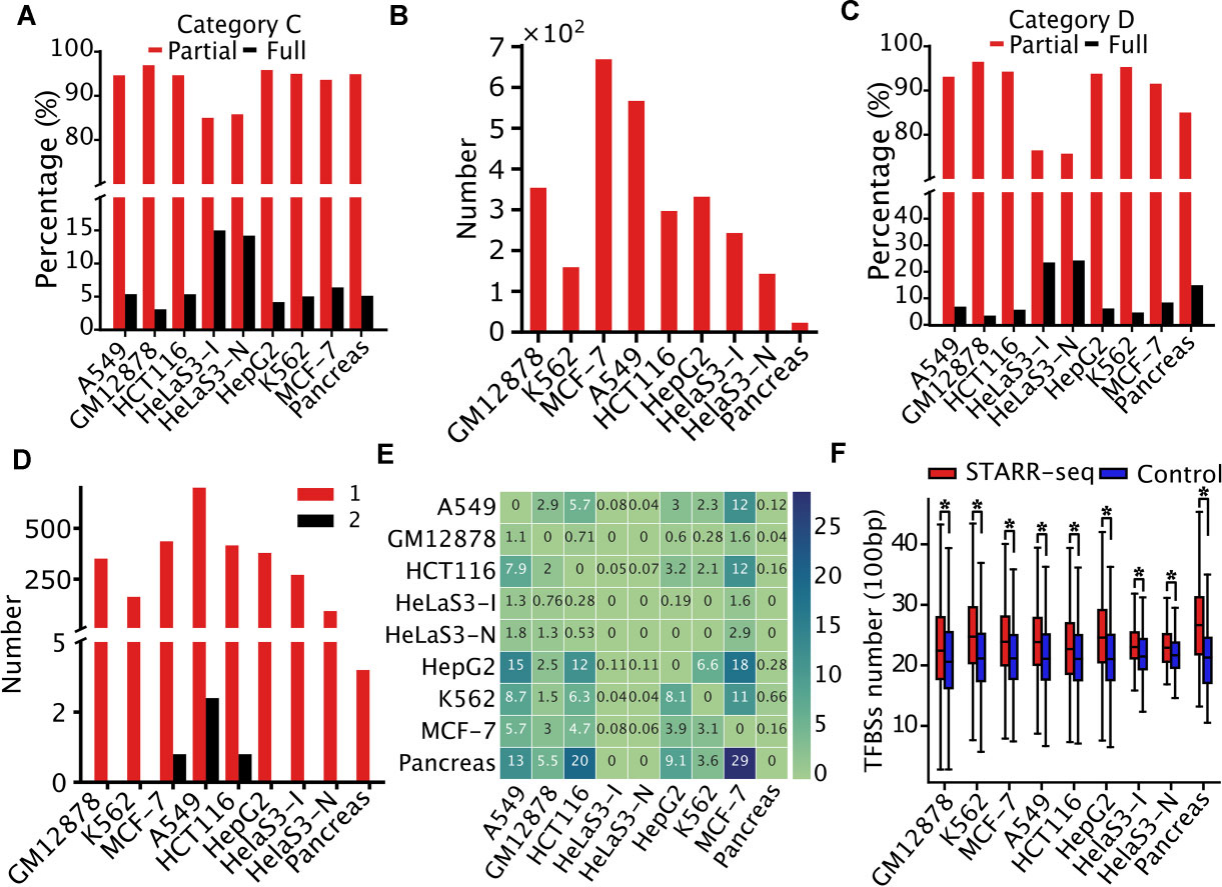
Comparison of STARR peaks and CRMs in the categories. (**A**) Percentages of the STARR peaks containing a full-length CRM and a partial CRM in category C in each cell line/tissue. (**B**) Number of CRMs in category C overlapping one STARR peak in each cell line/tissue; none of these CRMs overlaps more than one STARR peak. (**C**) Percentages of the STARR peaks containing a full-length CRM and a partial CRM in category D in each cell line/tissue. (**D**) Number of CRMs in category C overlapping one or at least two STARR peaks in each cell line/tissue. (**E**) Percentage of non-active CRMs in category D in a cell line/tissue (rows) that are active in other cell lines/tissues (columns). (**F**) Non-CRMs in category E contain a higher density of TFBS-like sequences than randomly selected non-CRMs with matched lengths and G/C content. *P< 0.001, two-tailed Mann–Whitney *U* test.

Category C comprises an average of 22.9% [C/(C + D + E)] of all STARR peak positions and 2.1% (C/(B + C)] of active CRM positions in the cell lines/tissues (Figure 1A and Supplementary Figure S10). Notably, pancreatic organoid assayed by CapSTARR-seq had the highest percentage (41.8%) of STARR peaks in category C among the nine cell lines/tissues (Supplementary Figure S10), suggesting that CapSTARR-seq might have the highest positive discovery rate. With an average median length of 500 bp, the STARR peaks in category C are somewhat shorter than the overlapping active CRMs with an average median length of 537 bp in pooled nine cell lines/tissues (Figure 2H) and in each cell line/tissue, except HeLaS3-I and HeLaS3-N cells, in which STARR peaks have uniform lengths with an average length of 1281 bp (Supplementary Figure S11), due to the aforementioned reason (31). Nonetheless, only a small proportion (3.1% in GM12878 to 15.0% in HeLaS3-I) of the STARR peaks include full-length active CRMs. While most (85–96.9%) of other STARR peaks overlap only parts of the active CRMs (Figure 4A), they overlap at least 50% of the full CRMs (see the ‘Materials and Methods’ section). On the other hand, although some of the CRMs in category C are longer than 1000 bp (Figure 2H and Supplementary Figure S11), all of them overlap only one STARR peak (Figure 4B). Consistently, CRMs in category C are generally much shorter than the average length (537 bp versus 1181 bp) of predicted CRMs. Thus, when lacking some sequences at the two ends, these relatively short CRMs might be still at least partially functional. Notably, the total length of STARR peaks in category C in HeLaS3-I cells (0.10% of the 85% genome regions) is twice as long as that in HeLaS3-N cells (0.05% of the genome regions) (Supplementary Figure S10), suggesting that treatment of cells with IFN-I response inhibitors could increase the sensitivity of STARR-seq methods. Moreover, using a longer (∼1500 bp) insertion sequence size in HeLaS3-I and HeLaS3-N cells largely increased the percentage (13.5–15%) of the STARR peaks covering the full overlapping active CRMs compared to using a shorter (∼500 bp) insertion size (3.1–6.2%) (Figure 4A).

### About 97.9% of predicted active enhancers might be missed by STARR peaks

As we indicated earlier, genes associated with category B (positions of active CRMs that do not overlap STARR peaks in the cell line/tissue) had similarly high gene expression levels to those associated with category C (positions of active CRMs that overlap STARR peaks in the cell line) (Figure 2A and Supplementary Figure S2); like genes associated with category C, genes associated with category B also had significantly higher expression levels (*P*< 5.03 × 10^−115^) than those associated with the other four categories (A, D, E and F) in pooled nine cell lines/tissues (Figure 2A) and in eight of the nine cell lines/tissues (Supplementary Figure S2). In contrast, the STARR-seq signal in category B was too weak (Figure 2B and Supplementary Figure S3) to form peaks. These results suggest that the predicted active CRMs in category B were likely to enhance the expression of the closest genes in their native chromatin, but were missed by STARR-seq assays, and thus were false negatives. As expected, category B is under strong evolutionary constraints (Figure 2C and Supplementary Figure S4), suggesting that the predicted active CRMs are likely true CRMs as we argued earlier (35,37). Consistently, like the active CRMs in category C, those in category B were also heavily modified by the active enhancer marks H3K27ac, H3K4me1 and H3K4me3, and had high CA, but were depleted of the repressive enhancer marks H3K9me3 and H3K27me3 (Figure 3 and Supplementary Figure S5). Moreover, these active CRMs are enriched for enhancer states (EnhG1, EnhG2, EnhA1, EnhA2, EnhWk and EnhBiv), but depleted of Het and ZNF/Rpts states in all the seven cell lines/tissues with ChromHMM annotations (Figure 2D and Supplementary Figure S6). They are also depleted of TEs in all the nine cell lines/tissues (Figure 2E and Supplementary Figure S7). These active CRMs have a similar level of complexity to that of CRMs in category A but are lower than those of CRMs in categories C and D (Figure 2F and Supplementary Figure S8). They have an average GC content of 41.6%, close to the average level of the human genome (41.0%) (Figure 2G and Supplementary Figure S9).

These predicted active CRMs comprise an average of 97.9% [B/(B + C)] of all predicted active CRM positions in a cell line/tissue (Figure 1A and Supplementary Figure S10). These results suggest that STARR-seq methods might have a false negative rate of up to 97.9%. Interestingly, these predicted active CRMs with an average median length of 1069 bp (Figure 1C and Supplementary Figure S1) are much longer than those in category C with an average median length of 537 bp in pooled nine cells/tissues (Figure 2H) and in each cell line/tissue (Supplementary Figure S11). The active CRMs in category B might likely be missed by STARR-seq methods that could only assess shorter (∼500 bp) sequences (Figure 1C and Supplementary Figure S1), and when cloned in the episomal expression vectors, the truncated forms of the long CRMs might no longer possess enhancer activities. Consistently, as shown in Figure 3 and Supplementary Figure S5, CA, H3K27ac, H3K4me1 and H3K4me3 signals of the CRMs in category B are much broader than those of the CRMs/STARR peaks in category C. Meanwhile, the signals of H3K27ac, H3K4me1 and H3K4me3 are not relatively depleted in the middle of the CRMs, indicating that accessible TFBSs are more broadly distributed along the CRMs. Thus, the truncated forms of active CRMs in category B might be no longer functional due to the lack of some critical TFBSs at the ends. Notably, the total length of STARR peaks in category B in HeLaS3-I cells is similar to that in HeLaS3-N cells (4.42% versus 4.45% of the 85% genome regions; Supplementary Figure S10), indicating that treatment of cells with IFN-I response inhibitors could only slightly reduce false negative rate (97.9% versus 98.8%) caused by the limitation of the short library insertion size. Moreover, the pancreatic organoid assayed by CapSTARR-seq had the highest false negative rate (99.9%) among the nine cell lines/tissues (Supplementary Figure S10), suggesting again the limitation of probes used in the CapSTARR-seq protocol to capture longer sequences.

### About 27.6% of STARR peaks are not active in their native repressive euchromatin

Both genes associated with category A (positions of non-active CRMs that do not overlap STARR peaks in the cell line/tissue) and genes associated with category D (positions of non-active CRMs that overlap STARR peaks in the cell line/tissue) had significantly lower (*P*< 1.76 × 10^−214^) expression levels than genes associated with categories B and C in pooled nine cell lines/tissues (Figure 2A) and in eight of the nine cell lines/tissues (*P*< 4.01 × 10^−6^) (Supplementary Figure S2), indicating that these predicted non-active CRMs, which together comprise an average of 88.5% of our predicted CRMs [(A + D)/(A + B + C + D)] in pooled nine cell lines/tissues, might not enhance the expression of their closest genes in the native chromosomal contexts. However, both categories A and D are under strong evolutionary constraints (Figure 2C and Supplementary Figure S4) as expected, suggesting that these predicted non-active CRMs are likely to be true CRMs as we argued earlier (35,37), but are in the non-active state in their native chromatin. Consistently, CRMs in both categories A and D were depleted of active enhancer marks H3K27ac, H3K4me1 and H3K4me3, and had low CA (Figure 3 and Supplementary Figure S5). However, CRMs in categories A and D differ in their repressive enhancer marks. Specifically, the former CRMs were moderately modified by both the Polycomb-mediated repressive mark H3K27me3 and the heterochromatin repressive mark H3K9me3, while the latter CRMs were heavily modified by H3K27me3 but moderately modified by H3K9me3 (Figure 3 and Supplementary Figure S5).

Moreover, STARR-seq signals in category A were too weak (Figure 2B and Supplementary Figure S3) to form STARR peaks. The CRMs in category A were depleted of enhancer states (EnhG1, EnhG2, EnhA1, EnhA2, EnhWk and EnhBiv), repressed Polycomb states (ReprPC and ReprPCWk), and Het and ZNF/Rpts states in all the seven cell lines/tissues with ChromHMM annotations (Figure 2D and Supplementary Figure S6). They were also depleted of TEs in all the nine cell lines/tissues (Figure 2E and Supplementary Figure S7) and had a similar level of complexity to that of CRMs in category B, but lower than those of CRMs in categories C and D (Figure 2F and Supplementary Figure S8). These CRMs have a lower average GC content (38.5%) than the human genome (41.0%) (Figure 2G and Supplementary Figure S9). In contrast, the non-active CRMs in category D are weakly enriched for enhancer states, including EnhG1, EnhG2, EnhA1, EnhA2, EnhWk and EnhBiv, and repressed Polycomb in all the seven cell lines/tissues with ChromHMM annotations (Figure 2D and Supplementary Figure S6). These CRMs are enriched for TEs in the cell lines (AS549, MCF-7, HCT116, HepG2 and K562) assayed by WG-STARR-seq and in pancreatic organoid assayed by CapSTARR-seq, but are depleted in GM12878 cells assayed by ATAC-STARR-seq and in HeLaS3-I and HeLaS3-N cells assayed by an improved STARR-seq method (Figure 2E and Supplementary Figure S7). The non-active CRMs in category D have a complexity higher than CRMs in categories A and B, but lower than those in category C in all the cell lines/tissues but HeLaS3-I cells (Figure 2F and Supplementary Figure S8). These CRMs have a higher average GC content (44.6%) than the human genome (41.0%) (Figure 2G and Supplementary Figure S9).

Furthermore, STARR-seq signals in category D were stronger than those in categories A and B, but weaker than those in category C in pooled cell lines/tissues and in seven out of nine cell lines/tissues (Figure 2B and Supplementary Figure S3), indicating that the resulting STARR peaks had strong enhancer activities in STARR-seq assays. These STARR peaks comprise an average of 27.6% [D/(C + D + E)] of all STARR peak positions in the nine cell lines/tissues, while the overlapping CRMs in category D only consist of an average of 0.3% [D/(A + D)] of non-active CRM positions (Figure 1A and Supplementary Figure S10). With an average median length of 500 bp, the STARR peaks in category D tend to be longer than the overlapping non-active CRMs with an average median length of 375 bp in pooled nine cell lines/tissues (Figure 2H) and in each cell line/tissue (Supplementary Figure S11). Still, only a small proportion (3.5% in GM12878 cells to 24.3% in HeLaS3-N cells) of the STARR peaks in category D include a predicted full non-active CRM. Although the majority (75.8–96.5%) of the other STARR peaks only contain parts of the overlapping non-active CRMs (Figure 4C), they overlap at least 50% of the full CRMs (see the ‘Materials and Methods’ section). On the other hand, although some of the non-active CRMs in category D are longer than 1000 bp (Figure 2H and Supplementary Figure S11), almost all of them overlap only one STARR peak with a few exceptions (Figure 4D). Therefore, when lacking some parts at the ends, the truncated forms of these relatively short non-active CRMs in category D might become active in episomal expression vectors presumably due to the absence of repressive enhancer marks as has been noted previously (22,34,46). Notably, the total length of STARR peaks in category D in HeLaS3-I cells (0.15% of the genome regions) is three times as long as that in HeLaS3-N cells (0.05% of the genome regions) (Supplementary Figure S10), suggesting that treatment of cells with IFN-I response inhibitors could increase the detection of short non-active CRMs in the cells. Moreover, STARR peaks in category D in HeLaS3-I cells comprise a higher percentage (37.3%) of identified STARR-seq than those in HeLaS3-N cells (30.2%) (Supplementary Figure S10). On the other hand, STARR peaks in category D in the pancreatic organoid assayed by CapSTARR-seq consist of the lowest percentage (13.0%) of identified STARR peaks among all the nine cell lines/tissues (Supplementary Figure S10). Thus, it appears that treatment of cells with IFN-I response inhibitors might increase and CapSTARR-seq might decrease the proportion of STARR peaks in category D. Moreover, using a longer (∼1500 bp) insertion sequence size in HeLaS3-I and HeLaS3-N cells largely increased the percentage (23.8–24.3%) of these STARR peaks covering the full overlapping non-active CRMs compared to using a short (∼500 bp) insertion size (3.5–6.2%) (Figure 4C).

We further reason that if non-active CRMs in category D in a cell line/tissue are masked by repressive histone marks as shown in Figure 3 and Supplementary Figure S5, then they could become active in certain other cell lines/tissues. To test this, we assessed overlaps between non-active CRMs in category D in a cell line/tissue and active CRMs/STARR peaks in category C (positions of active CRMs that overlap STARR peaks in the cell line) in other eight cell lines/tissues. As shown in Figure 4E, a varying portion of non-active CRMs in category D in a cell line/tissue is active in other cell lines/tissues. For example, 13%, 5.5%, 20%, 0.0%, 0.0%, 9.1%, 3.6% and 29% of non-active CRMs in category D in pancreatic organoid are active in A549, GM12878, HCT116, HeLaS3-I, HeLaS3-N, HepG2, K562 and MCF-7 cell lines, respectively (the last row in Figure 4E).

### About 49.5% of STARR peaks are enhancer-like sequences masked in heterochromatin

Genes associated with category E (positions of non-CRMs that overlap STARR peaks in the cell line/tissue) also had significantly lower expression levels than genes associated with categories B and C (*P*< 1.04 × 10^−3^) in pooled nine cell lines/tissues and in eight out of nine cell lines/tissues (Figure 2A and Supplementary Figure S2), suggesting that the STARR peaks in category E did not enhance the expression of the closest genes in their native chromatin. In contrast, STARR-seq signals in category E were stronger than those in categories A and B, but weaker than those in category C in pooled cell lines/tissues and in seven out of the nine cell lines/tissues (Figure 2B and Supplementary Figure S3), indicating that the resulting STARR peaks had strong enhancer activities in STARR-seq assays. Nonetheless, these STARR peaks were depleted of active enhancer marks H3K27ac, H3K4me1 and H3K4me3. They also had low CA and were heavily modified by the heterochromatin repressive mark H3K9me3, but not by the Polycomb-mediated repressive mark H3K27me3 (Figure 3 and Supplementary Figure S5). Moreover, category E is more likely evolutionarily neutral than the four CRM categories (A, B, C and D) (Figure 2C and Supplementary Figure S4, *P*< 2.23 × 10^−302^, K-S test), suggesting that the STARR peaks in category E might not be CRMs as we argued earlier (35,37). These STARR peaks are highly enriched for the ZNF/Rpts and Het states, but depleted of the enhancer states in six of the seven cell lines/tissues with ChromHMM annotations (Figure 2D and Supplementary Figure S6). They are enriched for LTR elements in all the nine cell lines/tissues (Figure 2E and Supplementary Figure S7) and have the lowest complexity due to their lack of TFBSs (Figure 2F and Supplementary Figure S8). These results suggest that these STARR peaks might largely reside in heterochromatin in the cell lines/tissues. These STARR peaks have the highest average GC content (47.1%) among categories A–E analyzed (Figure 2G and Supplementary Figure S9). Notably, STARR peaks in category E in HeLaS3-I cells have a lower average GC content (42.4%) than those in HeLaS3-N (46.2%) (Supplementary Figure S9).

The STARR peaks in category E comprise an average of 49.6% [E/(C + D + E)] of all STARR peak positions in the cell lines/tissues (Figure 1A and Supplementary Figure S10). With an average median length of 497 bp, the STARR peaks have similar length distributions to those in category C (Figure 2H) in pooled nine cell lines/tissues and each cell line/tissue (Supplementary Figure S11). These non-CRM sequences in heterochromatin likely have enhancer-like functions in episomal expression vectors, and thus these STARR peaks are false positives. Notably, HeLaS3-I cells have the lowest proportion of STARR peaks in category E (38.8%) among all the nine cell lines/tissues, including HeLaS3-N cells (39.3%) (Supplementary Figure S10), indicating that treatment of cells with IFN-I response inhibitors could considerably reduce the false positive rate that can be as high as 57.7% in K562 cells (Supplementary Figure S10).

### Enhancer-like sequences masked in heterochromatin might be evolved by genetic drift

Genes associated with category F (positions of non-CRMs that do not overlap STARR peaks in the cell line) had significantly lower expression levels than those associated with categories B and C (*P*< 5.03 × 10^−115^) in pooled cell lines/tissues and eight out of the nine cell lines/tissues (*P*< 3.38 × 10^−2^) (Figure 2A and Supplementary Figure S2). However, they had significantly higher expression levels than those associated with category E (*P*= 2.03 × 10^−27^) in pooled cells/tissues and eight out of the nine cell lines/tissues (*P*< 2.51 × 10^−2^) (Figure 2A and Supplementary Figure S2). As expected, like category E, category F is also more likely evolutionarily neutral than the four CRM categories (A, B, C and D) (Figure 2C and Supplementary Figure S4) (*P*< 2.23 × 10^−302^, K-S test), suggesting that non-CRMs in category F are not CRMs as we argued earlier (35,37). Consistently, non-CRMs in category F were depleted of the active enhancer marks H3K27ac, H3K4me1 and H3K4me3, and had negligibly low CA, but were moderately modified by the repressive enhancer marks H3K9me3 and H3K27me3 (Figure 3 and Supplementary Figure S5). These non-CRMs are likely in euchromatin. Then, why non-CRMs in category E showed enhancer activities in STARR-seq assays, while those in category F did not? A possible reason might be that no or weak evolutionary constraints on heterochromatin (47) render the STARR peaks in category E to become CRM-like sequences by genetic drift without causing deleterious effects as they are masked in heterochromatin. In contrast, unwanted CRM-like sequences in euchromatin such as those in category F can be deleterious and are subject to purifying selection (48). To test this hypothesis, we scanned these STARR peaks for the presence of putative TFBSs of TFs with known position weight matrices in the HOMER database (49) and found that they contain a significantly (*P*< 0.001) higher density of TFBS-like sequences than randomly selected non-CRM sequences with matched lengths and GC content (Figure 4F). It is highly likely that some of these TFBS-like sequences in category E might have enhancer activities in STARR-seq assays. The fact that the heterochromatin mark H3K9me3 heavily modifies category E but only moderately modifies category F (Figure 3 and Supplementary Figure S5) might at least partially explain why genes closest to the former category have lower expression levels in native chromatin than those closest to the latter category (Figure 2A and Supplementary Figure S2).

## DISCUSSION

Various strategies have been used to mitigate systematic errors and false positives and false negatives in the original STARR-seq method when applied to human cells. These strategies include using ATAC (24), histone marks (25) or microarray probes (26) to enrich potential active enhancers and increase insertion sequence size from ∼500 to ∼1500 bp (32), as well as using ORI as the core promoter of reporter and treatment of cells with IFN-I response inhibitors to reduce the systematic errors caused by plasmid transfection in human cells (31). However, we found that these current STARR-seq methods still suffer from very high false negative rates and false positive rates. More specifically, we found that these methods were only able to identify an average of 2.1% [C/(B + C)] of genomic positions of our predicted active CRMs (Figure 1A and Supplementary Figure S10). These identified active CRMs (category C) tend to have high complexity with dense TFBSs concentrated in their middle parts as evidenced by the depleted active histone marks in the center of the CRMs (Figure 3 and Supplementary Figure S5). They also tend to be short, with an average length slightly longer than that of overlapping STARR peaks. Although these STARR peaks may not contain fully overlapping active CRMs (Figure 4A), they are more likely to include the essential parts of the CRMs and thus are at least partially functional.

On the other hand, the current STARR-seq methods were unable to identify the remaining 97.9% [B/(B + C)] of genomic positions of our predicted active CRMs (category B) (Figure 1A and Supplementary Figure S10). Since we have excluded possible insulators, silencers and promoters, the vast majority of these predicted active CRMs are likely distal enhancers (35). In other words, current STARR-seq methods might have a false negative rate of up to 97.9%. Since most known enhancers are longer than 500 bp, for instance, enhancers in the VISTA database have an average length of 2049 bp (35), and our predicted CRMs have an average length of 1188 bp, while the plasmid (23,24) used in current STARR-seq methods can only accommodate an insertion size up to 500–1500 bp. Our predicted active CRMs in category B (average median length 1069 bp) tend to be much longer than the STARR peaks (average median length 500 bp); thus, they are too long to be fully inserted in the plasmids. Moreover, active epigenetic marks are more broadly distributed around the middle of these CRMs, suggesting that critical TFBSs can be located far away from their middle parts. Thus, their truncated forms that can be inserted in the plasmids may no longer be functional due to the lack of critical flanking. Our findings suggest a strategy to reduce the false negative rate. For example, using expression vectors that can accommodate longer insertion sequences might allow the detection of longer enhancers as also demonstrated in a recent study (32). However, since increasing insertion size from ∼500 bp (e.g. in A549, HCT116, HepG2, K562, MCF-7 and GM12878 cells) to ∼1500 bp (e.g. in HeLaS3-I and HeLaS3 cells) did not reduce false negative rate (Supplementary Figure S10), a vector that can accommodate much longer sequences (e.g. a few thousand bp) might be needed. Moreover, for some very long enhancers, different methods such as CRISPR interference (50,51) might be needed to validate them.

Equally importantly, we identified two types of STARR peaks that are located in repressive chromatin in the cell lines/tissues but display enhancer activity in episomal expression vectors. The first type is those in category D (Figure 1A and Supplementary Figure S10). They overlap our predicted non-active CRMs that are depleted of active enhancer marks but heavily modified by the Polycomb-mediated repressive mark H3K27me3 and moderately modified by the heterochromatin repressive mark H3K9me3 in their native chromatin. Interestingly, like our predicted active CRMs in category C, our predicted non-active CRMs in category D also tend to be short and even shorter than the overlapping STARR peaks (Figure 2H and Supplementary Figure S11). Therefore, like the STARR peaks in category C, the STAAR peaks in category D may also not contain fully overlapping non-active CRMs (Figure 4C); they are still likely to include essential parts of the CRMs and thus are at least partially functional in episomal vectors where repressive histone marks are not present. Consistently, we found that these CRMs/STARR peaks that were not active in their native chromatin in a cell line/tissue become active in the native chromatin in some other cell lines/tissues (Figure 4E). A similar phenomenon was also reported earlier (23,34). These relatively short CRMs may represent a part of repressive or inert enhancers in the cell lines/tissues as indicated by their ChromHMM states. Therefore, this type of STARR peaks are still highly valuable for characterizing all the regulatory elements encoded in the genome, although they are not active in native chromatin in the tested cell lines/tissues. They make up from 13.0% [D/(C + D + E)] of CapSTARR peaks in peracetic organoid to 37.3% of STARR peaks in HeLaS3-I cells treated with IFN-I response inhibitors. It appears that using longer insertion size (∼1500 bp) and IFN-I response inhibitors as in HeLaS3-I cells can facilitate identifying these relatively short CRMs (Supplementary Figure S10).

The second type of STARR peaks in repressive chromatin are in category E (Figure 1A). They overlap our predicted non-CRMs, and thus are likely false positives. As also reported previously (23,34), these STARR peaks are depleted of active enhancer marks and heavily modified by the heterochromatin repressive mark H3K9me3 but the Polycomb-mediated repressive mark H3K27me3. Thus, they are likely short CRM-like sequences masked in heterochromatin. These false positive STARR peaks contain a higher density of TFBS-like sequences than expected by chance (Figure 4F). These enhancer-like sequences might arise by genetic drift and are not wiped out by purifying selection (Figure 2C and Supplementary Figure S4), as they are located in heterochromatin (Figure 3 and Supplementary Figure S5), and thus are functionally dormant. Similar TFBS-like sequences formed by genetic drift in euchromatin would be wiped out by purifying selection to avoid detrimental effects. Therefore, these STARR peaks arise when the enhancer-like sequences that are largely selectively neutral and mostly masked in their native heterochromatin become active in episomal expression vectors. Ideally, this type of STARR peaks should be maximally reduced in a STARR-seq assay. Our findings suggest a strategy to reduce these false positive STARR peaks in heterochromatin, for example, by depleting heterochromatin, or by enriching naked DNA using ATAC, DNase digestion or formaldehyde-assisted isolation of regulatory elements, when preparing insertion sequences from genomic DNA. Indeed, we showed that ATAC-STARR-seq (24) had a lower proportion (44.2%) of STARR peaks in category E than did WHG-STARR-seq (23) (48.4%) that did not enrich naked DNA. Moreover, we found that treating cells with IFN-I response inhibitors can even more effectively reduce this type of STARR peaks (38.8%). Nonetheless, a more efficient method for depleting heterochromatin during insertion sequence preparation is needed to further reduce the proportion of this type of STARR peaks. One possible method is to combine an ATAC or DNase digestion-based method with a ChIP step targeting marks such as H3K4me1 or H3K27ac for active and poised enhancers.

## Supplementary Material

lqad085_Supplemental_Files

## Data Availability

The data underlying this article are available in Zenodo, at https://doi.org/10.5281/zenodo.8320401.

## Notes

Present address: Pengyu Ni, Department of Molecular Biophysics & Biochemistry, Yale University, New Haven, CT 06520, USA.
