# Supplementary material for: Underlying causes for prevalent false positives and false negatives in STARR-seq data
Source: NAR Genom Bioinform. 2023 Sep 22;5(3):lqad085. doi: 10.1093/nargab/lqad085 (PMC10516709; doi:10.1093/nargab/lqad085)
Supplement: lqad085_Supplemental_Files [file lqad085_supplemental_files.zip › Supplementary Information_R1_8_24_2023.pdf]

Supplementary Information

Underlying causes for prevalent false positives and false negatives in STARR-seq data

Pengyu Ni[§], Siwen Wu, Zhengchang Su[*]

Department of Bioinformatics and Genomics, the University of North Carolina at Charlotte, Charlotte, NC, 28223, USA

* To whom correspondence should be addressed. Tel: +01-704-687-7996; Fax: +01-704-687-8667; Email: zcsu@uncc.edu

[§]Current address: Department of Molecular Biophysics & Biochemistry, Yale University, New Haven, CT, 06520, USA

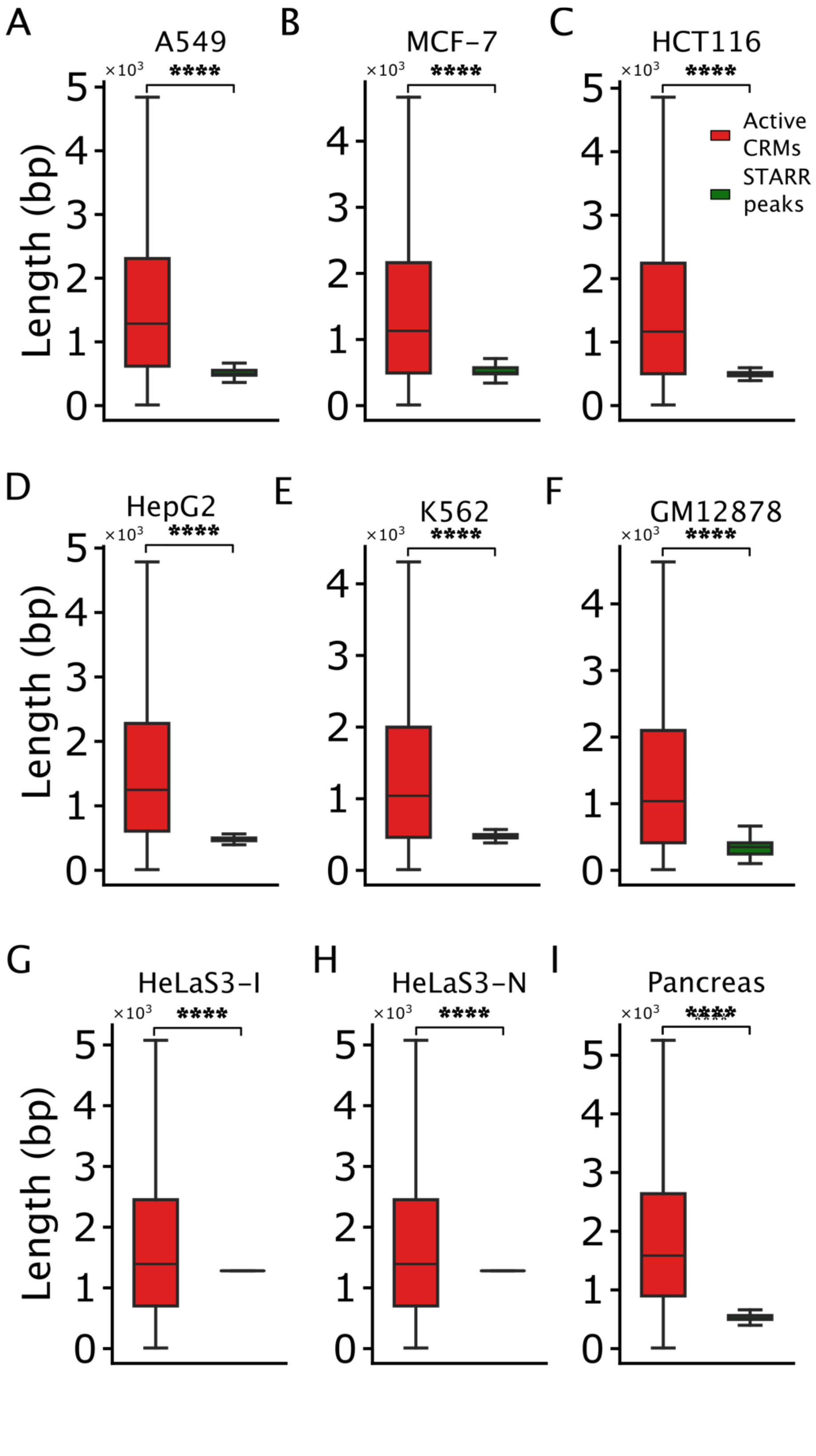

Supplementary Figure 1. Distributions of the lengths of STARR peaks and of predicted active CRMs in each cell line/tissue. **** $P < 10^{-04}$, two-tailed Mann-Whitney U test.

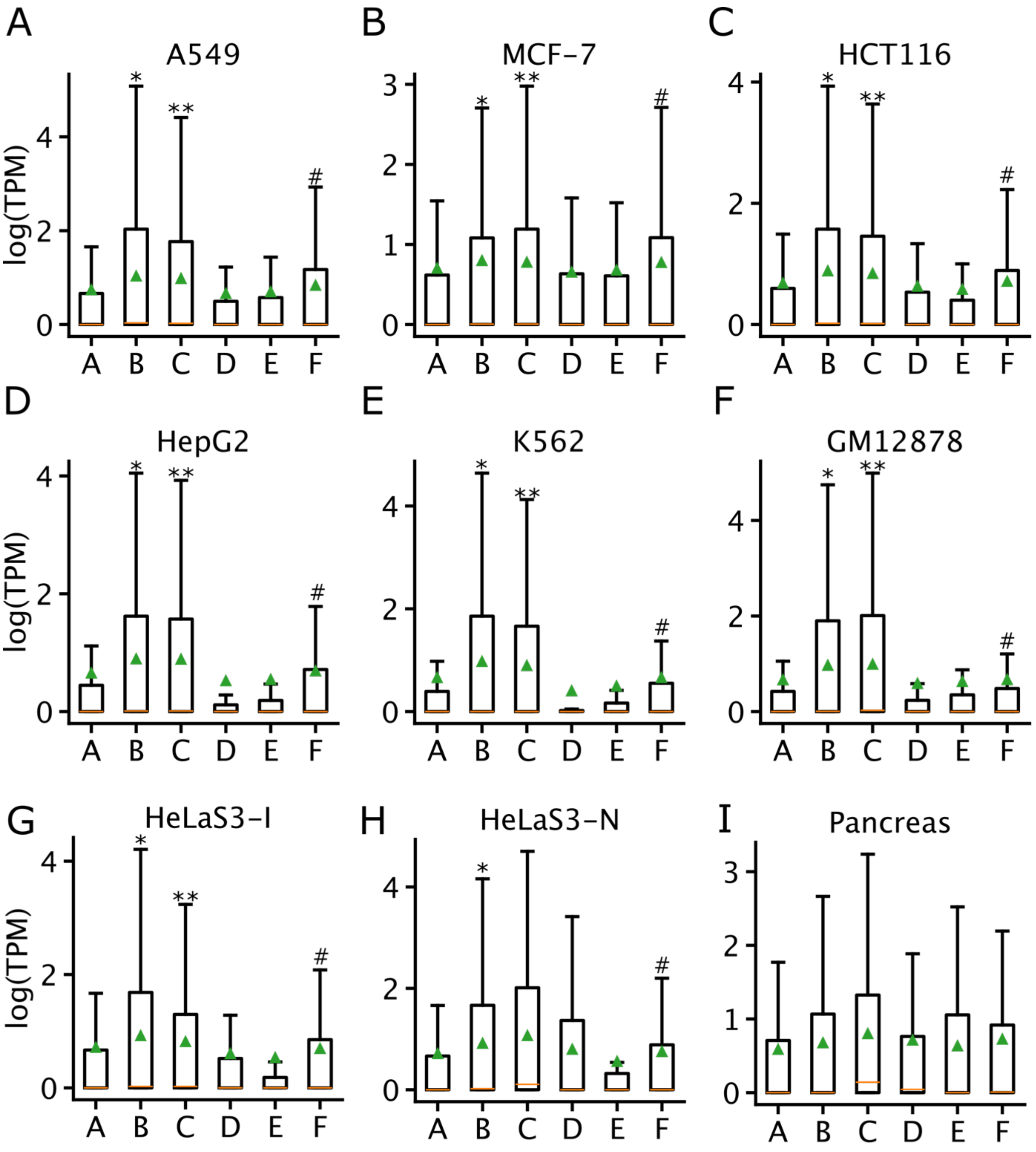

Supplementary Figure 2. Expression levels of closest genes to STARR peaks and to predicted active CRMs, non-active CRM and non-CRMs in the six categories in each cell line/tissue. *$p<0.05$, comparison between genes associated with category B and those associated with categories A, D, E, and F. **$p<0.05$, comparison between genes associated with category C and those associated with other five categories. $^{\#}p<0.05$, comparison between genes associated with category F and those associated with category E. All tests were done using two-tailed Mann-Whitney U test.

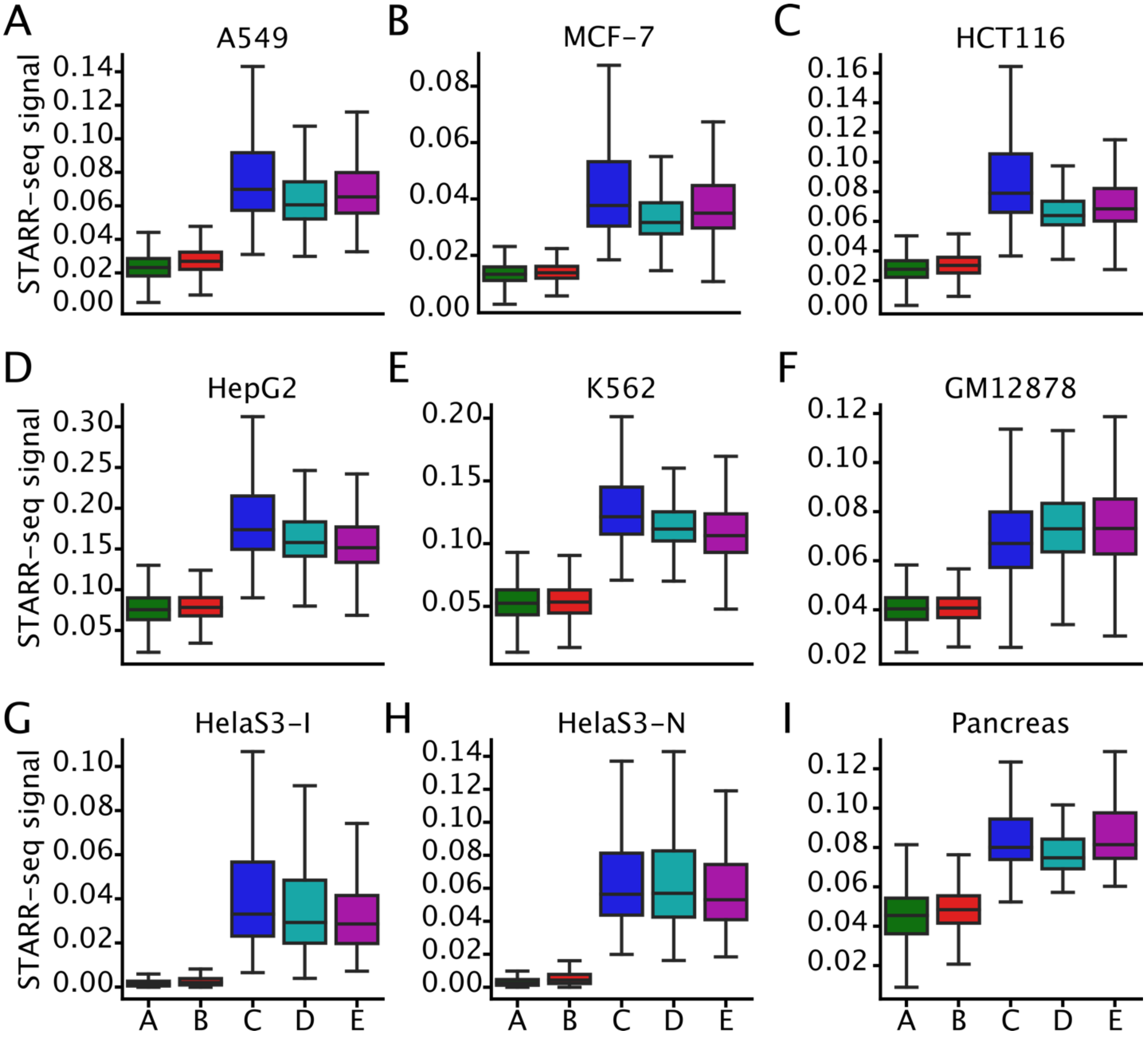

Supplementary Figure 3. Boxplot of STARR-seq signal strengths in categories A~E regions in each cell line/tissue.

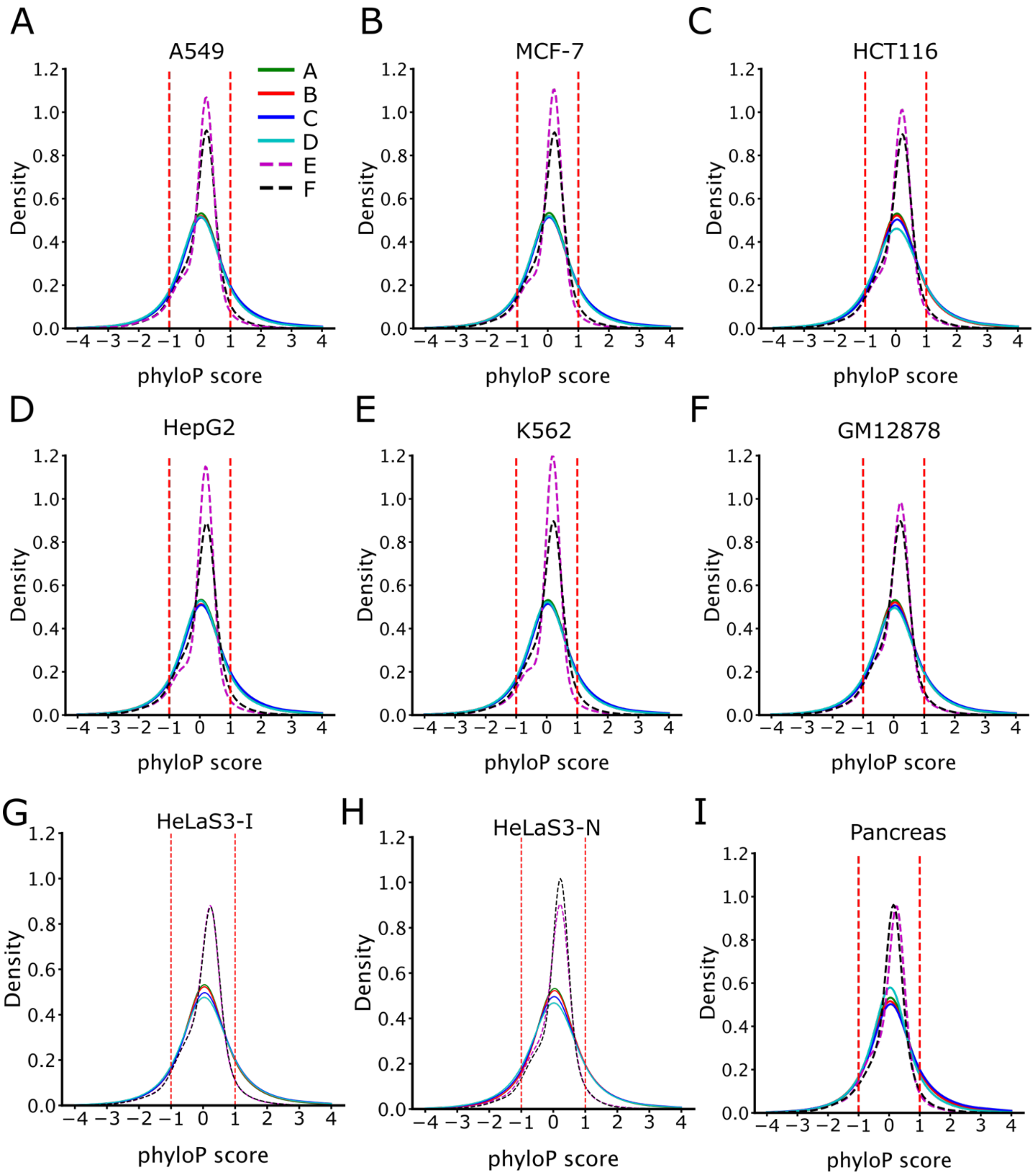

Supplementary Figure 4. Distributions of phyloP scores of nucleotide positions of the six categories in each cell line/tissue.

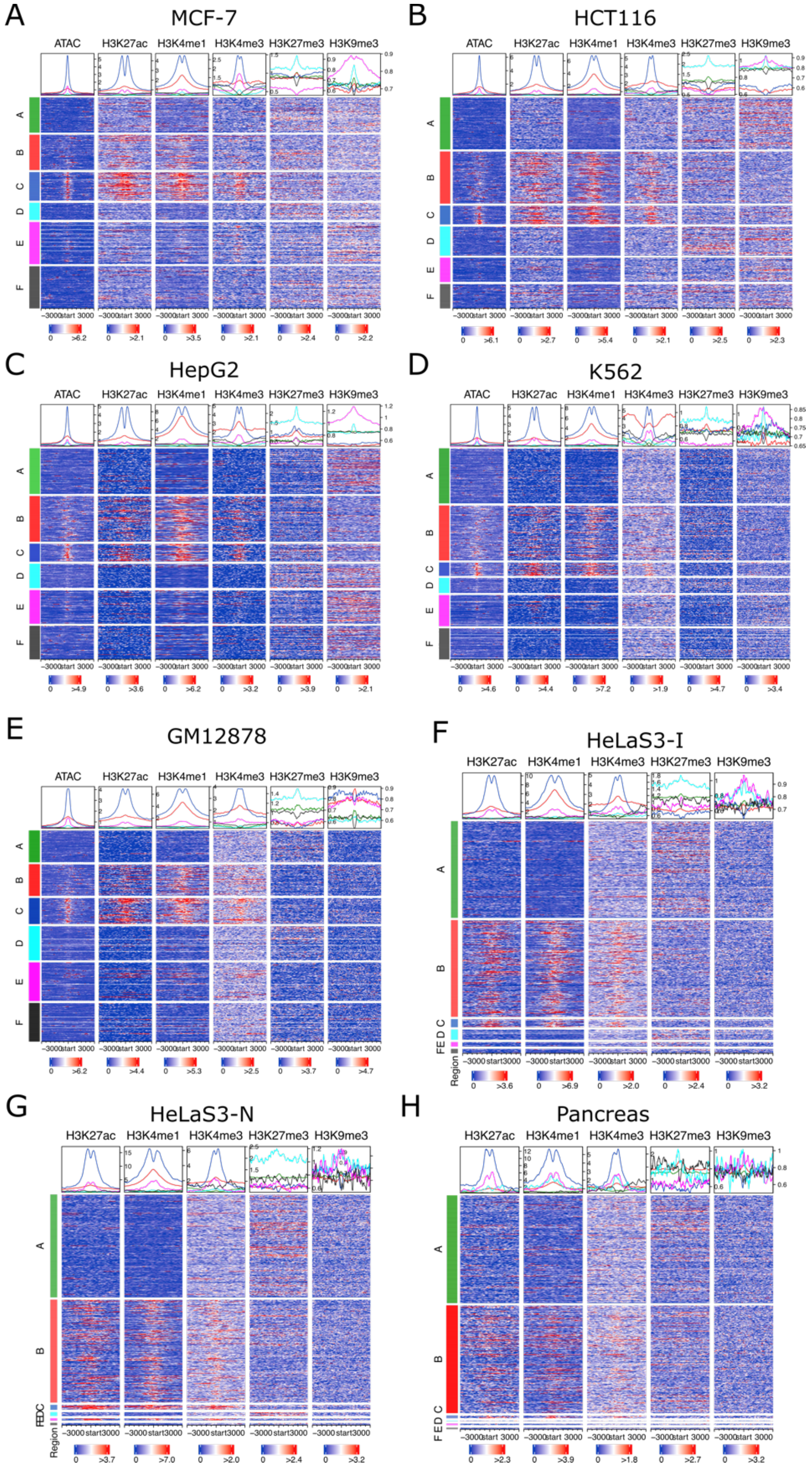

Supplementary Figure 5. Heat maps of various chromatin signals in the six categories in each cell line/tissue. The heatmap shows the mean in each window in each sequence and the density plot shows the mean of each window position across all the sequences in the same category. The color code for the categories in the density plot above each column is the same as in the heatmaps.

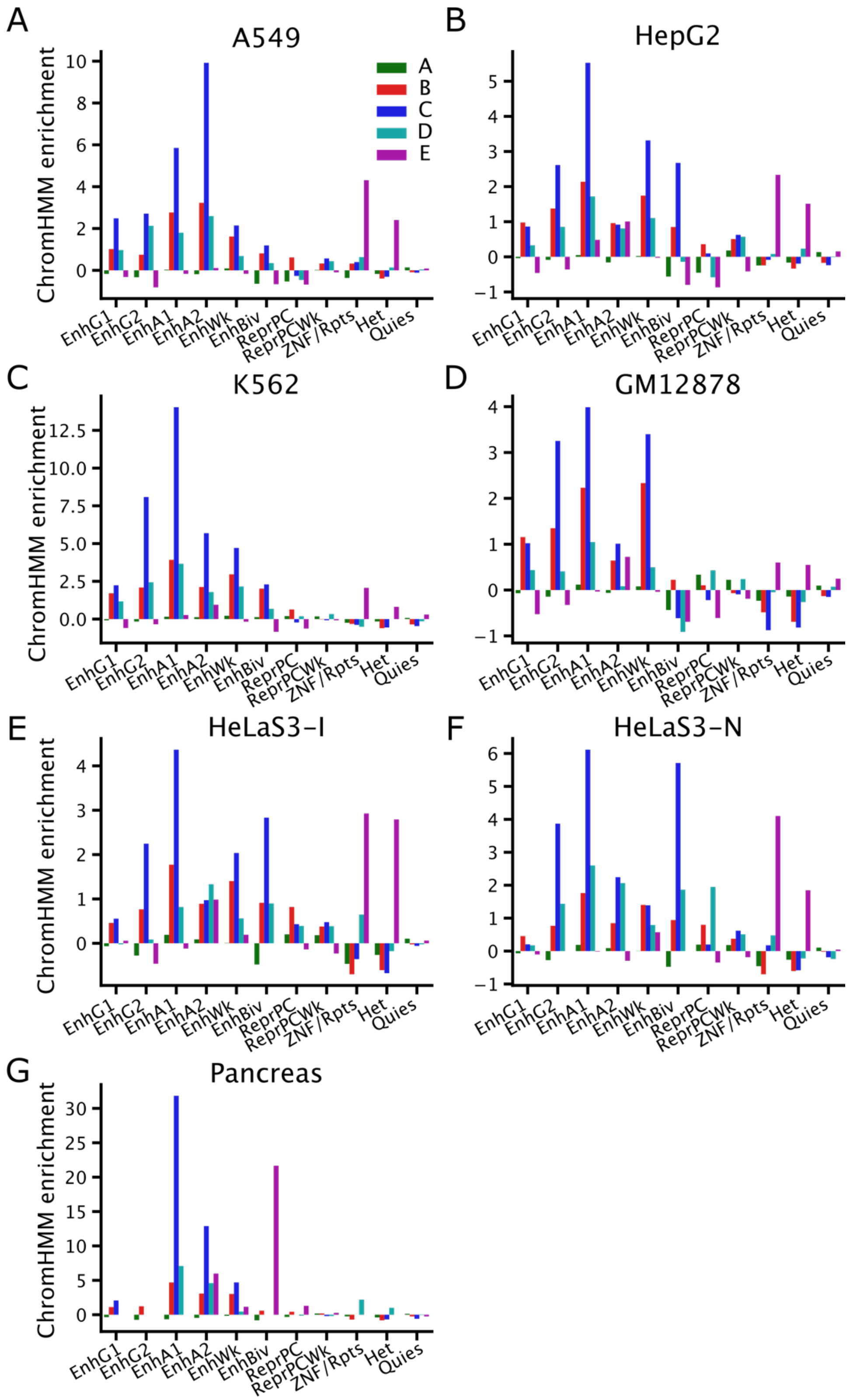

Supplementary Figure 6: Enrichment of the ChromHMM states in categories A-E in each cell line/tissue.

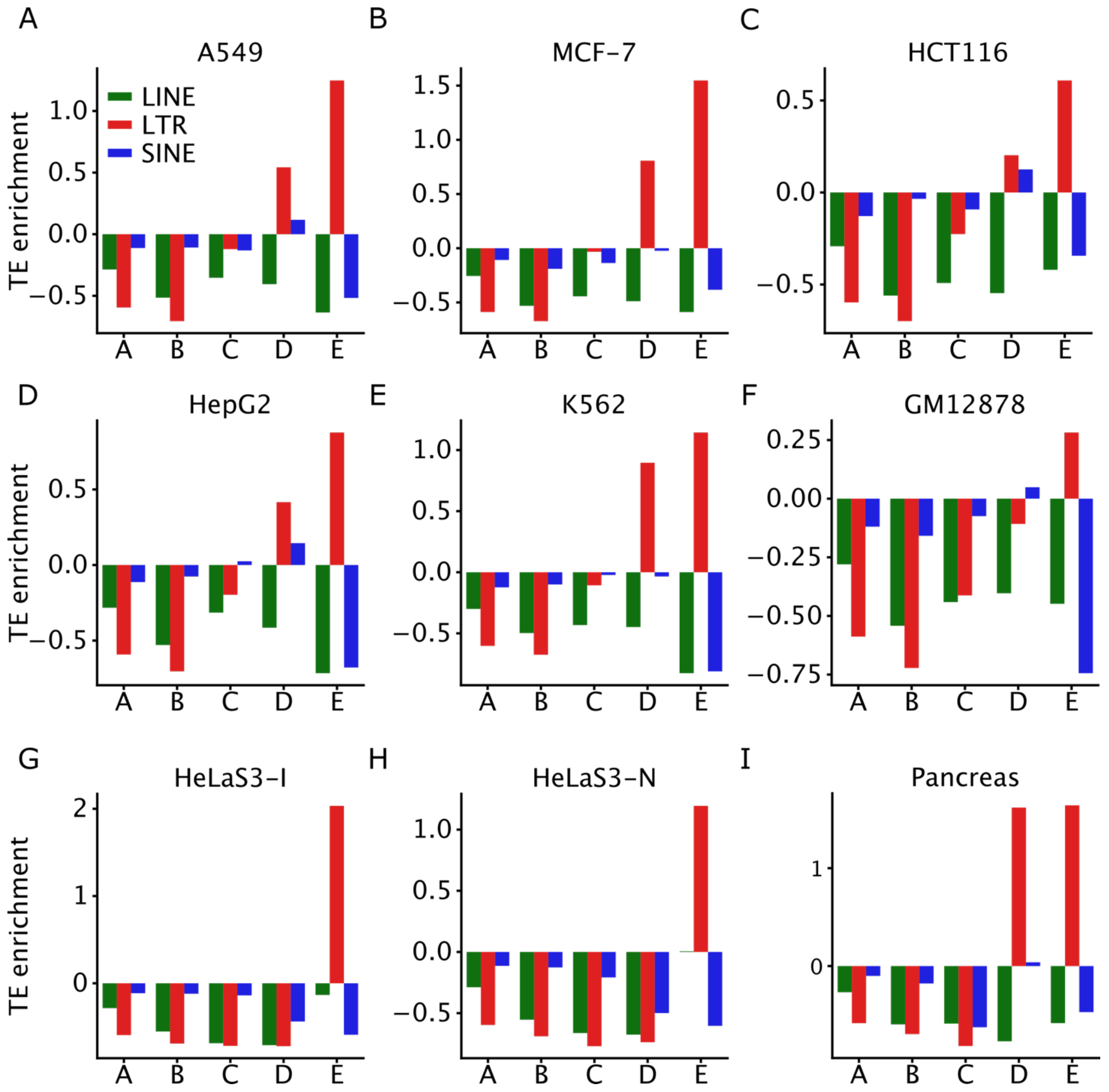

Supplementary Figure 7: Enrichment of transposable elements in categories A-E in each cell line/tissue.

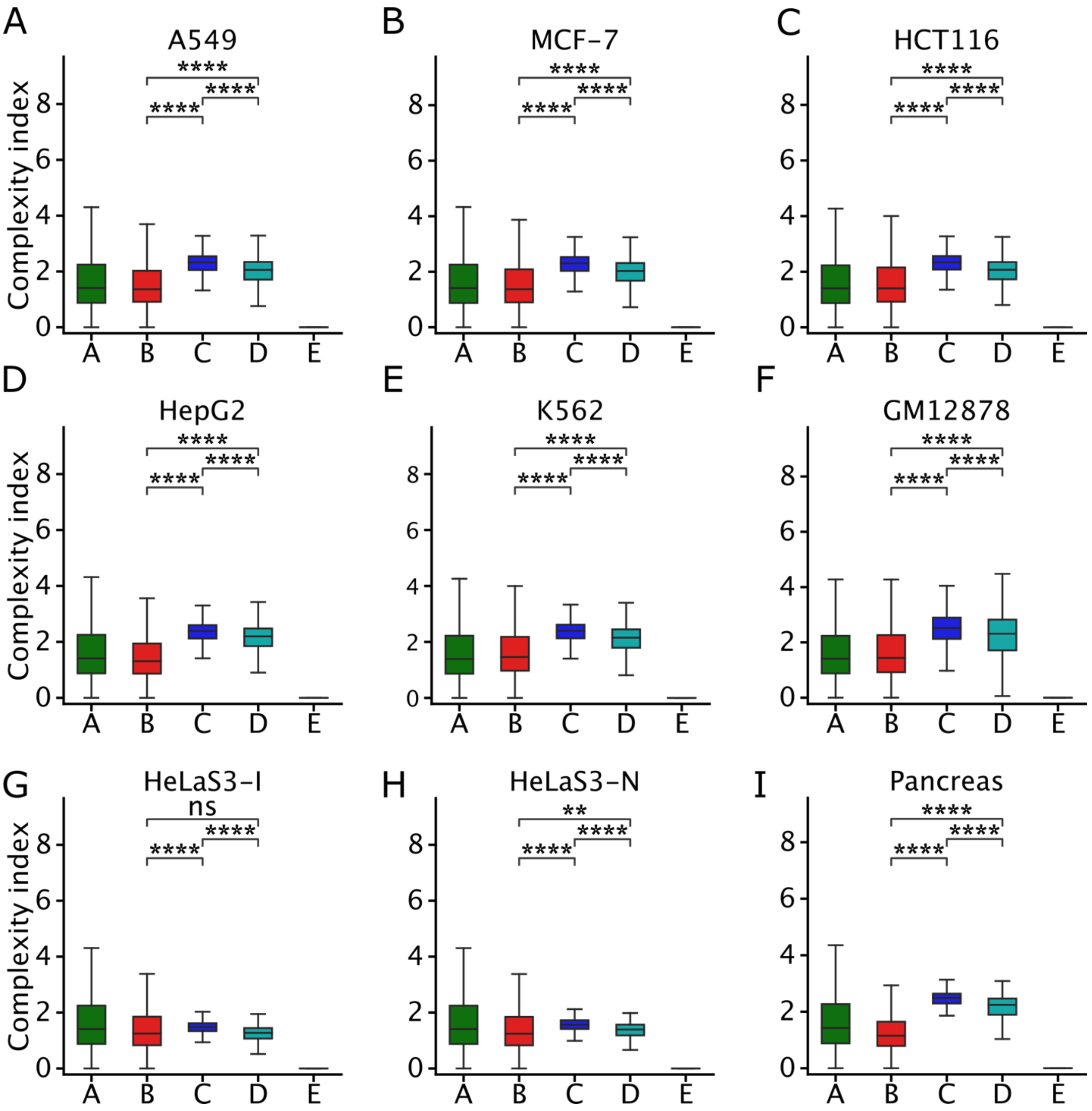

Supplementary Figure 8: Boxplots of complexity index of the CRMs/STARR-peaks in categories A-E in each cell line/tissue.

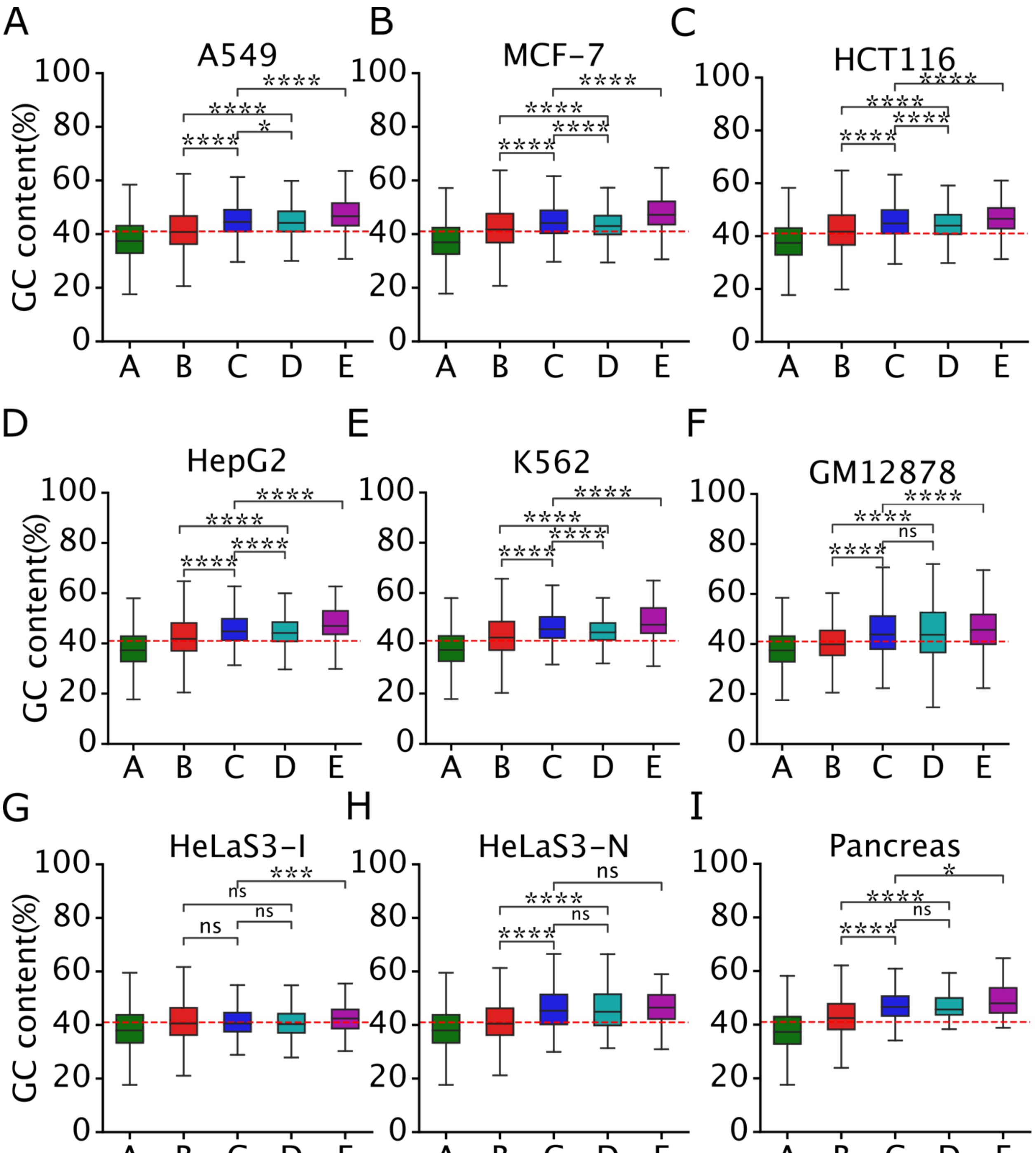

Supplementary Figure 9: Boxplots of GC content of categories A-E in each cell line/tissue. The dotted horizontal line in each plot indicates the mean GC content of 41% in the human genome.

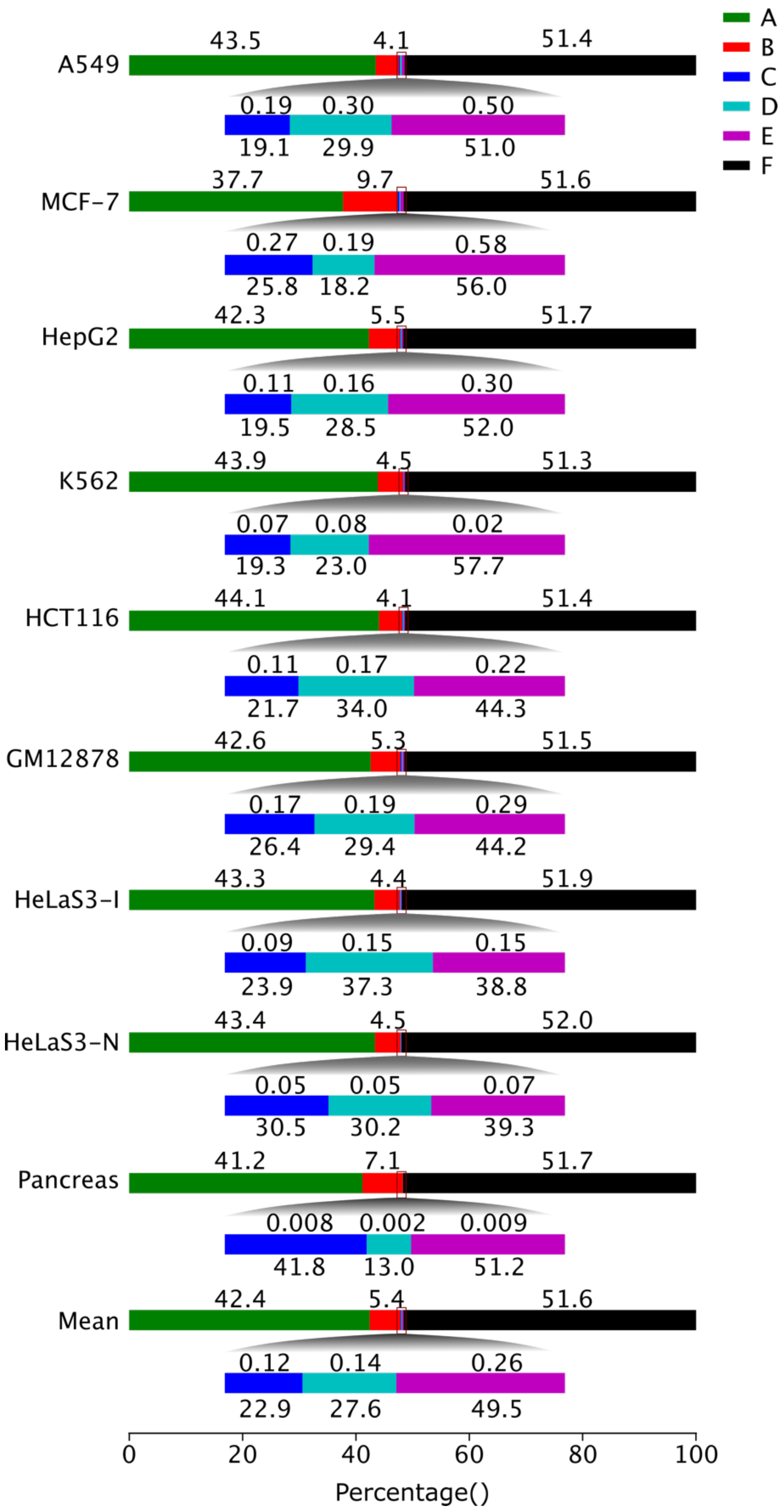

Supplementary Figure 10. Cartoon showing the percentages of sizes of the six categories in the genome in each cell line/tissue, as well as their means in nine cell lines/tissues. The blow-up views show STARR peak categories C, D and E, and the number under a box is the relative percentage of the corresponding category. The number above a box is the percentage of the corresponding category in the 85% of the genome. The axis at the bottom indicates the proportion of each category in the 85% of the genome.

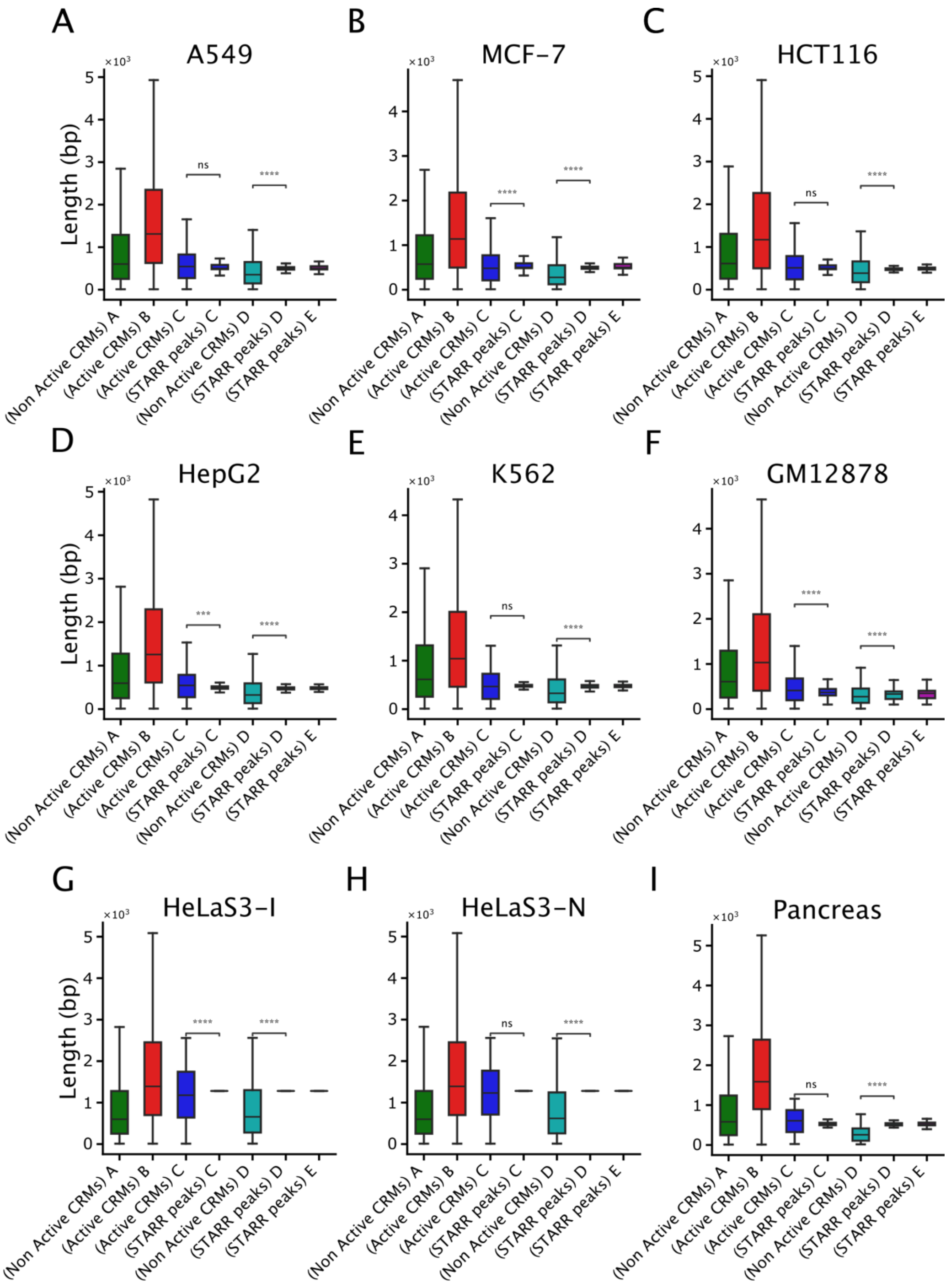

Supplementary Figure 11. Distributions of the lengths of predicted active CRMs and of non-active CRMs in categories A~D, and of STARR peaks in categories C~E in each cell line/tissue.
